# Clustering Digestive Tract Tumors Using Transcriptomic and Mutation Data

**DOI:** 10.3390/cancers18091427

**Published:** 2026-04-30

**Authors:** Dwayne G. Tally, Polina Bombina, Jake Reed, Jeffrey Kinne, Lynne V. Abruzzo, Kevin R. Coombes, Zachary B. Abrams

**Affiliations:** 1 Department of Informatics, Indiana University, Bloomington, IN 47408, USA; 2 Department of Biostatistics Data Science, Epidemiology Georgia Cancer Center, Augusta University, Augusta, GA 30912, USAkcoombes@augusta.edu (K.R.C.); 3 Department of Oncological Sciences, Huntsman Cancer Institute, Academic Medical Center, Health University of Utah, Salt Lake City, UT 85112, USA; 4 Department of Computer Science, Indiana State University, Terre Haute, IN 47809, USA; 5 Department of Pathology, Medical University of South Carolina, Charleston, SC 29425, USA; abruzzo@musc.edu; 6 Institute for Informatics, Data Science & Biostatistics, Washington University, St. Louis, MO 63110, USA

**Keywords:** TCGA, digestive tract cancer, clustering, mutation, genomics, transcriptomics, head and neck cancer, esophageal cancer, gastric cancer, pancreatic cancer, colon cancer, rectal cancer

## Abstract

Background: Digestive tract cancers, like most other cancers, are usually categorized based on cell or tissue of origin. Molecular clustering based on the transcriptome often produces the same classification. Methods: We developed a new method, Newmanization, to reduce underlying tissue signals from transcriptomic analysis. To test our method, we downloaded data on 1635 samples of digestive tract cancers from The Cancer Genome Atlas. The available data includes transcriptomic data by RNA-Seq, as well as binary mutation allele frequency data by whole exome sequencing. We compared, using silhouette widths and visualization by dimension reduction plots, the effectiveness of Newmanized transcriptome and mutation data to separate digestive tract cancers. Results: The Newmanized transcriptome clusters have clearer separation and larger average silhouette widths. Feature analysis of each cluster for Newmanized transcriptomic data and mutation data revealed that clusters determined with Newmanized data contained more mRNAs present at higher frequencies than clusters defined by mutation data. Conclusions: This suggests that the Newmanized method holds great potential for advancing personalized transcriptomic medicine.

## Simple Summary

Like most cancers, digestive tract tumors are classified by the tissue of origin (stomach, colon, etc. Clustering tumors based on gene expression patterns yields the same groups. Using data on six kinds of cancer, we first tested the hypothesis that clustering based on patterns of mutated genes would yield better groupings and highlight “driver” mutations that could be targeted by new treatments. That hypothesis did not work as well as expected. So, we developed a new method, the “Newman bank test” to explore the gene expression changes caused by the driver mutations. Our new approach discovered clusters that crossed tissue boundaries and could more clearly be linked to targetable mutations. This new approach could contribute to a better molecular classification of cancers and could potentially suggest better treatments for individual patients.

## 1. Introduction

Cluster analysis has been an integral part of the analysis of gene expression data since its earliest days [1]. In general, the goal of these analyses has been to discover subtypes of already known cancer types, usually characterized by the tissue or anatomic site of origin. Beginning in the mid-2000s, as The Cancer Genome Atlas (TCGA) project accumulated data on cancers of different types, many of their initial publications applied clustering to find subtypes within individual cancer types, using either single- or multi-omic data sets. They typically reported roughly three to six subtypes of every cancer (ovarian, three or four [2]; colorectal, four [3]; breast, four [4]; lung squamous cell, four [5]; clear cell renal cell, four [6]; glioblastoma, six [7]).

The implicit assumption underlying this class of applications of clustering to gene expression data is that different types of cancer are each characterized by a small set of “drivers” comprising genomic (somatic mutations and copy number changes) and epigenetic lesions. These lesions then impose a dominant “signal” on the gene expression patterns that reflect the specific ways that some critical pathways and hallmarks of cancer are broken in those cancer types. The mutations, in particular, have precipitated the growth of “personalized medicine” in cancer by providing therapeutic targets that are detectable in individual tumors.

Once TCGA grew large enough to contain data on cancer types from many different organs, researchers began applying clustering as part of their “pan-cancer” analyses [8]. If that implicit assumption was correct, one might expect the pan-cancer clusters to cross the boundaries between organs, since (a) the gene expression in each cancer should reflect the mutation patterns and (b) many of the same cancer driver genes are mutated in different kinds of cancer. Instead, it soon became clear that the “type of cancer” signal was unlikely to be the strongest part of the gene expression profile.

For example, Hoadley and colleagues [9] reported that: “We performed molecular clustering using data on chromosome-arm-level aneuploidy, DNA hypermethylation, mRNA, and miRNA expression levels and reverse-phase protein arrays, of which all, except for aneuploidy, revealed clustering primarily organized by histology, tissue type, or anatomic origin.” Wu and colleagues [10] found that “the pan-cancer analysis results show that the cancers of different tissue origins are generally grouped as independent clusters, except squamous-like carcinomas.” Taskesen and colleagues drew similar conclusions from their multi-omic clustering study [11]. The same result has been found in single omic studies, including methylation profiles [12], mutational signatures [13], and in proteomic patterns from The Cancer Proteomic Atlas (TCPA) [14].

The dominance of the signal from the cell or tissue of origin persisted even when one is restricted to the expression of smaller sets of genes. We previously performed a clustering analysis across TCGA cancer types by using the gene expression patterns of a representative subset of transcription factors. We found that the primary driver of cluster formation was the cell or tissue of origin [15]. We obtained similar results when clustering samples based on the expression of a representative set of microRNAs [16]. Careful perusal of the t-SNE plots in those papers (and in many of the papers cited above) suggests that it is the cell, rather than the tissue, of origin that dominates. For example, non-small-cell lung cancer has long been separated into two histological subtypes: adenocarcinoma or squamous cell carcinoma. These subtypes were treated as different primary types by TCGA, and they cluster separately in pan-cancer analyses. Moreover, the squamous cell lung cancers tend to cluster relatively close to, but not overlapping, squamous cell cancers from other sites, including head-and-neck or cervix. Esophageal cancer also has two histological subtypes. Esophageal adenocarcinomas tend to cluster with stomach adenocarcinoma, and esophageal squamous cell carcinomas tend to cluster with head-and-neck squamous cell tumors [17].

One way to get around the dominance of the cell-of-origin signal can be found in a report from Akbani and colleagues, who clustered TCGA samples using reverse-phase protein array (RPPA) data [18]. Tissue of origin was found to be a strong driver of clustering when the data were processed using standard normalization. However, this effect was significantly reduced after applying tissue-specific median centering to remove the signal from tissue types. A potential problem with this approach, however, is that we have no idea exactly what is being thrown away along with the tissue-of-origin signal. Important properties that are common to the entire set of cancers may also be discarded.

In this paper, we introduce the “Newman banked statistic”, which we compute using a “bank” of normal control samples based on the tissue of origin for each type of cancer. We standardize the gene expression patterns in tumor samples using the observed mean and standard deviation of the normal bank. Using this method, we have control over our modifications to the full transcriptomic profile, ensuring that we only remove the signal corresponding to average normal tissue expression. The resulting data for each gene is then measured in how many standard deviation units it is away from the normal mean. By setting a threshold in such units, we can convert the standardized expression data to binary data, with “0” representing “in the normal range” and “1” representing “outside the normal range”. We refer to the entire process of standardization and dichotomization as “Newmanization”. We suspect that Newmanized transcriptomic data may give a clearer picture of the functional effects of important drivers on gene expression by automatically filtering out the weaker effects of passenger mutations.

Our goal here is to test two hypotheses. First, we hypothesize that clustering using the binary mutation data should produce clusters based on mutation patterns rather than cell of origin. Second, we hypothesize that clustering using the binary Newmanized transcriptomic data will produce clearer and more interpretable clusters. To test these hypotheses, we will use six cancers from anatomic sites along the digestive tract (head-and-neck, esophagus, stomach, pancreas, colon, and rectum) from the collection of TCGA data. As with most cancers, digestive tract cancers are usually categorized based on cell type and location [19]. However, colon and rectal cancers are almost indistinguishable based on their usual transcriptomic profiles [3,15]. These relationships may reflect only the normal expression patterns in the cell of origin. Our hypotheses are that mutation patterns, especially as reflected in Newmanized transcriptomic data, will perform better at identifying meaningful molecular subtypes that cut across the boundaries defined by anatomical sites. Digestive tract cancers provide an ideal context to test this approach, given their diversity in tissue types and mutation patterns. This diversity should reveal whether unsupervised mutation clustering or Newmanized clustering can uncover meaningful molecular subtypes independent of cell of origin. If successful, this study could pave the way for advances in precision oncology by offering insights into cross-tissue cancer subtypes and identifying novel therapeutic targets.

## 2. Methods

### 2.1. Data

We obtained transcriptomic and mutation allele frequency data from TCGA. The transcriptomic data contains diseases associated with colon (COAD), rectum (READ), pancreas (PAAD), stomach (STAD), esophagus (ESCA), and head and neck (HNSC). Table 1 shows the number of samples per digestive tract disease type. The transcriptomic data contains continuous measurements of 20,531 features (i.e., genes) on samples from 1635 patients. The mutation data set is binary (wild-type or mutated) for the same 1635 patients but only has 563 features (i.e., potentially mutated genes).

### 2.2. Software

All analyses were performed using version 4.1.1 of the R statistical software environment. We used a variety of public R packages: NewmanOmics version 1.1.2, Mercator version 1.1.7 [20], and Polychrome version 1.5.4 [21]. Clustering was performed using the implementation of hierarchical clustering with Ward’s linkage rule in the *hclust* function in the stats package from base R. Cluster assignment quality was assessed quantitatively using the silhouette width [22,23]. Visualization was performed using the Mercator interface to the t-SNE algorithm in version 0.17 of the Rtsne R package [24]. All R packages are available from the Comprehensive R Archive Network (CRAN) [25].

### 2.3. Newman Bank Test

We used our *NewmanOmics* R package to process the TCGA transcriptomic data. The underlying logic of the Newman bank statistic is derived from D. Newman’s 1939 studentized range statistic [26]. We converted the expression of gene g by standardizing it using a cohort (or “bank”) of normal control samples:

$$\nu (g)=\frac{g-Mean_{Bank}(g)}{SD_{Bank}(g)}$$

Intuitively, if the observed value in a cancer sample is far enough away from the mean expression of the normal bank when measured in units defined by an *independent* estimate of standard deviation, then it is (potentially) an outlier. The expression pattern of these outlier genes may yield a better way to cluster samples.

A critical property of Newman’s statistic is that it requires an independent estimate of the standard deviation. It is not enough to compare cancer samples to a cohort of normal samples by simply estimating the usual gene-by-gene standard deviation from the set of normal samples. That restriction is especially relevant when the normal set is small (such as the pancreatic cancer data set from TCGA, which contains only four normal samples). Our innovation is to calculate this “independent” estimate by leveraging the repeated observation that the standard deviation of gene expression is a smooth function of the mean [27,28]. This function can be estimated using just the normal bank of samples by applying the loess algorithm [29]. Thus, by taking a loess fit across the entire set of (normal) gene expression profiles, we can calculate an estimated standard deviation for each gene, $SD_{Bank}(g)$, that borrows strength from all genes with similar means.

### 2.4. Dichotomization and Clustering

After standardizing the usual transcriptomic data using the loess-smoothed estimate of standard deviation, we converted the resulting matrix into binary, based on a cutoff value measured in standard deviations. Specifically, any gene whose expression was within three standard deviations of the mean was coded as “0”, and any gene whose expression was more than three standard deviations above or below the mean was coded as “1”.

We then applied the Mercator package to both the Newmanized transcriptome data and the mutation data. Since both data sets are binary, we used the Jaccard distance metric [30]. The Jaccard similarity, $S_{J}$, between two binary vectors is defined as

$$S_{J}=\frac{N_{11}}{N_{01}+N_{10}+N_{11}},$$

where $N_{ij}$ counts the number of times a value equals i in the first vector and j in the second vector. Then, the Jaccard distance is defined as $D_{J}=1-S_{J}$. One advantage of using the Jaccard distance instead of other measures of difference between binary vectors is that it ignores the non-informative “0-0” matches when events (like mutations) are relatively rare and/or their absence is less important than their presence.

We applied hierarchical clustering to six select clusters from the Jaccard distance matrices, with one cluster for each known disease category. We used silhouette widths to measure the intrinsic quality of clustering [22,23]. To compute the silhouette width for sample i, you first define a(i) to be the mean distance from that sample to the other samples assigned to the same cluster. You also compute the mean distance from sample i to every other cluster and define b(i) to be the minimum of the mean distances to other clusters. Then, you take the silhouette width to be

$$sw(i)=\frac{b(i)-a(i)}{max(a(i),b(i))}.$$

The values of the silhouette width lie between −1 and +1. When the silhouette width is positive, then the clustering is good. When the score is negative, the cluster assignment is poor. Kaufman and Rouseeuw suggested using the mean silhouette width (over all samples) as a quantitative tool to choose the best set of cluster assignments. We also used the *Polychrome* R package [21] to generate standard colors in order to display the clusters in a t-SNE plot [24], for purposes of visualization. In all applications of t-SNE, we used the default parameters of the *tsne* function in the *Rtsne* package.

### 2.5. Interpretation

We used summary statistics to estimate the frequency of each event (mutated gene or abnormally expressed gene) in each cluster. In order to identify genes that were substantially different between clusters, we used both gene-by-gene chi-squared tests on the binary Newmanized data and gene-by-gene analysis of variance (ANOVA) on the continuous ν-values. Finally, we performed gene enrichment analysis using ToppGene (https://toppgene.cchmc.org/, Accessed on 12 April 2026) [31].

## 3. Results

### 3.1. Comparing Clusters from Usual and Newmanized Transcriptomic Data

**Figure 1:**
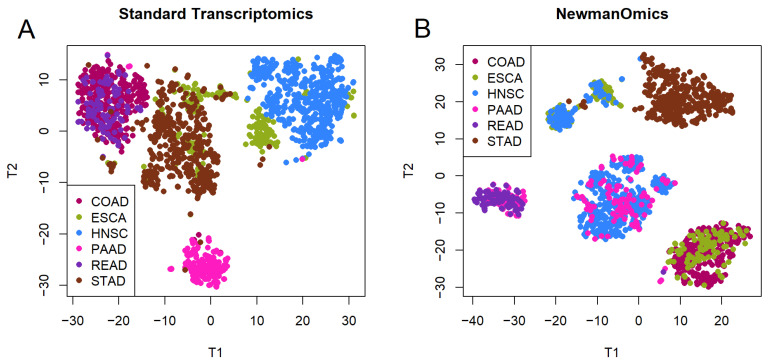
t-SNE comparison between (**A**) standard and (**B**) Newman transcriptomic data. Colors relect the tissue or cell of origin. The axis labels ‘T1’ and ‘T2’ are arbitrary names for the coordinates produced when applying the t-SNE algorithm to the data for dimension reduction and visualization.

As shown in Figure 1A, the usual transcriptomic clusters are defined mainly by the cell type or tissue of origin. There are essentially four clusters, consisting of pancreas (PAAD), colorectal (COAD, READ), stomach (STAD), and head-and-neck (HNSC). The esophageal tumors (ESCA) cluster either with stomach (if adenocarcinomas) or head and neck (if squamous cell cancers). However, clusters learned from the binary Newmanized transcriptome data are not defined by tissue (Figure 1B), though they show a similar degree of separation to the usual transcriptomic clusters. In this plot, stomach tumors are mostly clustered apart from all other DT cancers. All the other diseases are split between at least two clusters, and each of the four additional clusters combines multiple tumor types. One cluster contains esophagus and head and neck; a second contains esophagus and colon; a third contains head and neck and pancreas; and the fourth contains pancreas and rectal.

### 3.2. Comparing Newman Transcriptomic and Mutation Allele Frequency

**Figure 2:**
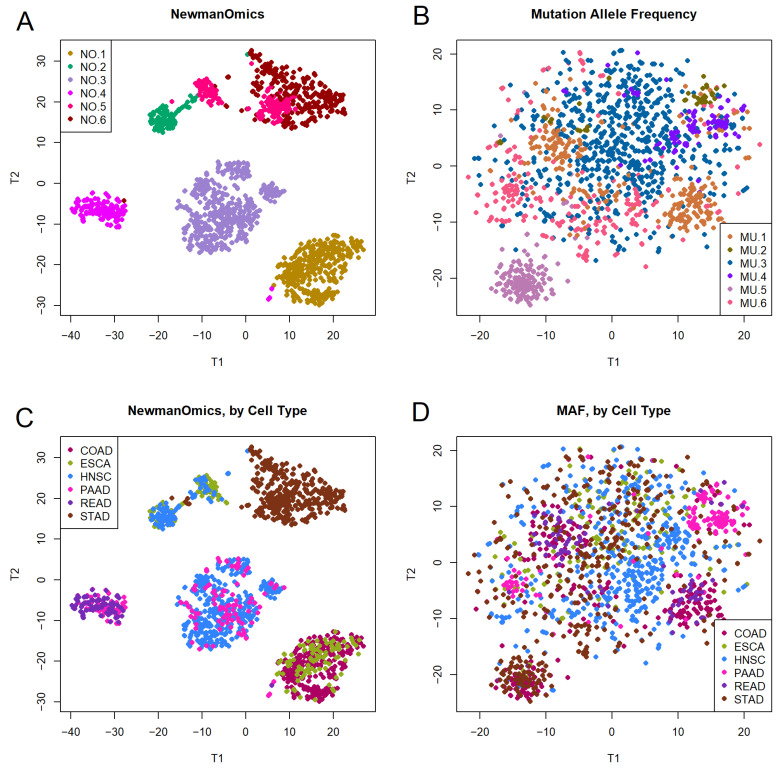
Default t-SNE graphical analysis of binary Newmanized transcriptomic (**A**,**C**) and mutation allele frequency (**B**,**D**) data based on hierarchical clusters (**A**,**B**) or colored by disease type (**C**,**D**). Both (**A**,**B**) are colored by hierarchical clusters; (**C**,**D**) are colored by known diseases. The prefix ‘NO’ is used to describe clusters found by clustering binary Newmanized data; the prefix ‘MU’ is use to identify clusters found by clustering binary mutation data; the numbers 1–6 are assigned arbitrarily. (**A**) shows Newmanized transcriptomic data. (**B**) shows mutation allele frequency hierarchical clusters. (**C**) shows the transcriptomic clusters but colored by the disease type. (**D**) shows the mutation allele frequency clusters but colored by disease type.

The next figure (Figure 2) is a four-part panel that uses t-SNE to visualize hierarchical clusters and tissue types between Newman transcriptomic and mutation allele frequency clustering. The t-SNE plots in Figure 2A,C are the same underlying plots derived from the Newmanized transcriptome data. Figure 2A colors the points based on unsupervised hierarchical clustering, while Figure 2C uses colors to indicate the tissue of origin, and is identical (by intention) to Figure 1B. The six hierarchical clusters in Figure 2A match one’s expectations from the structure of the t-SNE plot. Meanwhile, Figure 2B,D show the corresponding plots based on the binary mutation data. The boundaries between clusters in Figure 2B are quite blurry, with very little in the form of recognizable, separable clusters. Figure 2D uses different colors to show the tissue of origin of the tumor samples. There is some indication of overlap between hierarchical clusters and tissue type, but it is not as strong as that seen with the transcriptomic data in Figure 2A.

### 3.3. Silhouette Width

**Figure 3:**
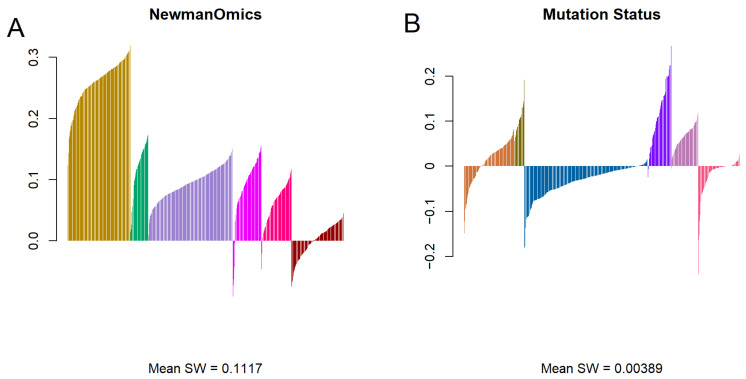
Silhouette width plots for hierarchical clustering of (**A**) Newmanized transcriptomic and (**B**) mutation allele frequency data. Negative values identify samples that are closer to a different cluster than the one to which it was assigned. Colors are the same as in Figure 2A,B to denote clusters discovered from the data.

Figure 3 visualizes the silhouette width plots for hierarchical clusters using either (A) Newmanized transcriptomic or (B) mutation data. The silhouette width of the Newmanized data visually shows a higher rate compared to the silhouette width of the mutation data. The mean silhouette width of the Newmanized transcriptomic data is substantially higher (μ = 0.0828) than the mean silhouette width of the mutation data (μ = 0.0039), suggesting that the Newmanized transcriptomic data produces better cluster assignments than the mutation data. The plot for the Newmanized transcriptomic data shows that almost every sample was categorized into the correct cluster, except for the sixth (brown) cluster where many samples had negative values. Mutation silhouette widths are mostly negative except for the second (dark olive), fourth (purple), and fifth (lavender) clusters. This indicates that most samples in the other clusters could have been categorized better.

### 3.4. Common Features Associated with Hierarchical Clusters

Table 2 lists the top five features most frequently abnormally expressed or mutated in each cluster from each of the binary data sets. Panel A (columns 1–3) lists mRNA features when clusters are found from Newmanized transcriptomic data. Panel B (columns 4–6) lists mRNA features when clusters are created from mutation data. Panel C (columns 7–9) lists mutated genes when clusters are found from Newmanized data. Panel D (columns 10–12) lists mutated genes when clusters are created from mutation data. Features listed in Panel A are present at a much higher percentage than those in any other panel.

### 3.5. Chi-Squared Tests

Mutated gene features in Panel D (clustered by mutation) of Table 2 are typically at higher frequencies than those in Panel C (clustered by Newmanized transcriptome). While it is not terribly surprising that the most frequently mutated genes occur at higher rates when the clusters are defined by mutation patterns, one must take special care when trying to interpret these results. For example, *TP53* is the most commonly mutated gene across the entire combined data set, where it is mutated in 62.1% of all samples. It is listed as one of the five most commonly mutated genes in cluster 6 of the mutation hierarchical data, but it is only present in 10.9% of those samples. This observation suggests that *TP53* mutations are more notable for their absence in these samples than their presence. To address this issue, we performed chi-squared tests for each gene in each cluster to determine which gene mutations are present at higher rates than would be predicted by their overall mutation rate.

Mutated genes associated with NewmanOmics clusters:

- NO.1 (279 COAD, 91 ESCA cases): *APC* (Overall: 22.1%; Here: 56.5%, $\chi^{2}=373.9$), *BRAF* (Overall: 5.0%; Here: 12.7%; $\chi^{2}=64.1$), *FAT4* (Overall: 14.4%; Here: 21.4%; $\chi^{2}=50.6$), plus ten others.
- NO.2 (91 READ, 68 PAAD cases): *KRAS* (Overall: 19.8%; Here: 54.1%; $\chi^{2}=193.5$), *NRAS* (Overall: 1.5%; Here: 6.3%; $\chi^{2}=36.4$).
- NO.3 (404 STAD cases): *ARID1A* (Overall: 11.4%; Here: 25.8%; $\chi^{2}=115.0$), *CDH1* (Overall: 3.4%; Here: 9.6%; $\chi^{2}=64.4$), *ACVR2A* (Overall: 5.0%; Here: 10.8%; $\chi^{2}=63.2$), plus ten others.
- NO.4 (173 HNSC, 54 PAAD cases): *CDKN2A* (Overall: 9.8%; Here: 19.7%; $\chi^{2}=83.5$), *HRAS* (Overall: 2.0%; Here: 5.6%; $\chi^{2}=35.7$), *FAT1* (Overall: 11.9%; Here: 19.7%; $\chi^{2}=30.4$) *CASP8* (Overall: 5.0%; Here: 10.2%; $\chi^{2}=29.4$).
- NO.5 (91 HNSC, 90 ESCA cases): *NFE2L2* (Overall: 3.3%; Here: 8.0%; $\chi^{2}=30.6$), *TP53* (Overall: 62.1%; Here: 79.1%; $\chi^{2}=29.3$).
- NO.6 (235 HNSC, 49 PAAD cases): *NOTCH1* (Overall: 9.1%; Here: 15.3%; $\chi^{2}=29.1$).

Mutated genes associated with mutation clusters:

- MU.1 (150 COAD, 62 READ, 76 other cases): *APC* (Overall: 22.1%; Here: 74.5%; $\chi^{2}=498.8$).
- MU.2 (27 PAAD; 12 STAD; 21 other cases): *SMAD4* (Overall: 8.9%; Here: 98.3%; $\chi^{2}=579.5$).
- MU.3 (318 HNSC, 184 STAD, 136 ESCA, 91 other cases): *TP53* (Overall: 62.1%; Here: 77.8%; $\chi^{2}=175.0$).
- MU.4 (74 PAAD, 42 HNSC, 25 other cases): *KRAS* (Overall: 19.8%; Here: 58.2%; $\chi^{2}=350.1$), *CDKN2A* (Overall: 9.8%; Here: 44.0%; $\chi^{2}=200.5$).
- MU.5 (81 STAD, 56 COAD, 22 other cases): *ACVR2A* (Overall: 5.0%; Here: 40.3%; $\chi^{2}=438.8$), *ZFHX3* (Overall: 6.2%; Here: 45.3%; $\chi^{2}=436.1$) *ARID1A* (Overall: 11.4%; Here: 62.3%; $\chi^{2}=428.6$) plus more than a hundred others.
- MU.6 (90 HNSC, 88 STAD, 70 other cases): none.

**Figure 4:**
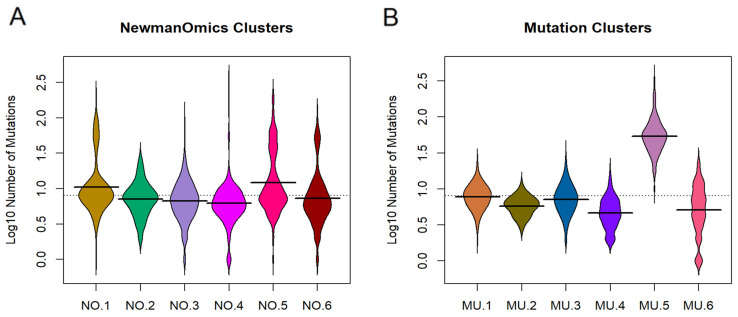
Mutation burden in clusters defined by (**A**) Newman transcriptomic data and (**B**) mutation data. Cluster colors are the same as in previous figures.

The large number of genes associated with cluster MU.5 suggests that those samples may simply have more mutations than other cases. To test this idea, we plotted the distribution of the number of mutated genes per sample within clusters (Figure 4). Most samples have fewer than ten mutated genes, but the samples in cluster MU.5 have closer to 100 mutations each. These samples appear to be distributed across Newmanized clusters NO.1, NO.5, and NO.6, which have more associated mutated genes than other Newmanized clusters. We also note that there are 25 samples that have no mutated genes; all 25 are assigned to cluster MU.6 (which has no significantly associated genes). In the Newmanized clusters, these samples are scattered loosely across at least five of the six clusters.

### 3.6. Gene Enrichment

In order to understand the expression differences between the six clusters using the Newmanized transcriptome data, we performed two analyses. First, we performed gene-by-gene ANOVA on the continuous ν-values. Second, we performed gene-by-gene chi-squared tests on the binary values. The complete results of both analyses are presented in an Excel spreadsheet (Supplemental Table S1). In both cases, the false discovery rate (FDR) associated with nominal *p*-value cutoffs, p<0.01, satisfied FDR<0.01. In order to select genes strongly associated with individual clusters, we restricted tests to the set of genes for which p<0.01 for both the ANOVA F-test and the chi-squared test, and which were found to be abnormally expressed (i.e., outside the normal range) in at least 10% of all cases. This produced a list of 4677 genes. Each gene was allocated to the cluster that contributed the largest percentage of its cases to the set with abnormal expression. (By cluster, this procedure yielded 2719 genes for NO.1, 352 for NO.2, 104 for NO.3, 353 for NO.4, 652 for NO.5, and 505 for NO.6.) These gene sets were uploaded to the ToppGene website for gene enrichment analysis, with parameters set to return at most 40 annotations per major ToppGene category. A full set of the enrichment tables produced by the website can be found in an Excel spreadsheet (Supplemental Table S2).

**Figure 5:**
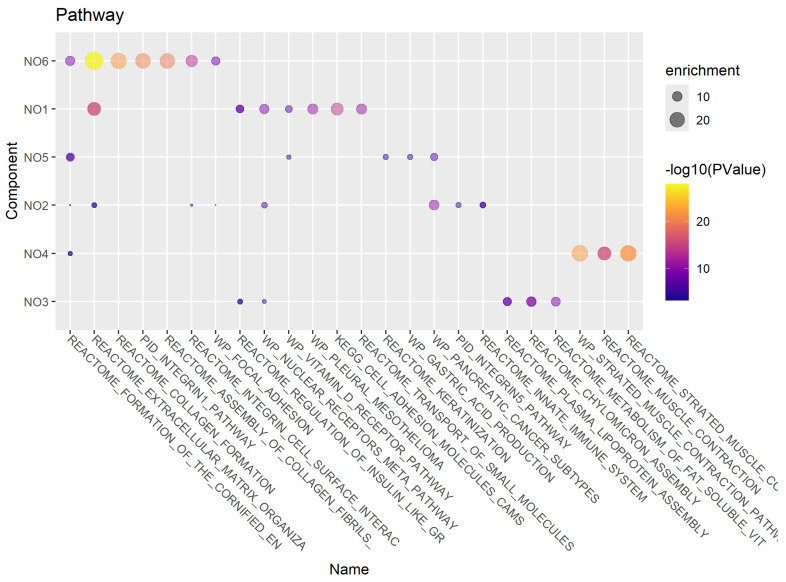
Summary of gene enrichment analyses for pathways, by NewmanOmics clusters.

For visualization, we restricted to the five most significant annotations per cluster, along with any annotation that was associated with more than one cluster. The results for enriched pathways are shown in Figure 5. We note that multiple clusters are associated with the extracellular matrix, along with collagens, integrins, and adhesion.

#### 3.6.1. Differences Between Colon and Rectal Cancer

Next, in order to better understand the differences between cluster NO.1 (COAD and ESCA) and cluster NO.2 (READ and PAAD), we selected all genes significantly associated with either of those clusters for which they had also either the highest or lowest average expression of ν-values. We used this set of 1159 genes for gene enrichment analysis with ToppGene. Here, the roles of the extracellular matrix, adhesion, collagens, and integrins were even more prominent. Among the pathway associations, 30 of the top 40 and 19 of the top 20 associated annotations were from this set of pathways. The same was true of four of the top six gene ontology molecular function annotations, four of the top eight for biological process, and six of the top 10 for cellular component.

#### 3.6.2. Differences Between Subtypes of Esophageal Cancer

In NewmanOmics clustering, the esophageal cancers are split across two clusters, NO.1 (COAD and ESCA) and NO.5 (HNSC and ESCA). We compared this split to the known split into squamous cell carcinoma and adenocarcinoma (Table 3).

While there is a statistically significant association of adenocarcinoma with Newmanized cluster *NO.1* ($\chi^{2}=22.344$, $p=2.28\times 10^{-9}$), this association is far from perfect. To better understand which features are driving the Newmanized clustering, we performed a similar gene enrichment analysis comparing NO.1 and NO.5, using 376 genes. We found significant differences that could be linked to two specific chromosomal cytobands. First, nine histones and nine HLA Class II genes in the HLA region on cytoband 6p21.3 were expressed at higher levels in cluster NO.5 compared to cluster NO.1. There were also associations with autoimmunity, T cell expression and receptors, and CTLA expression. Second, three APOBEC3 genes in cytoband 22q13.1 were also expressed at higher levels in cluster NO.5.

## 4. Discussion

Our first finding (Figure 1A) confirms previously published results that suggest that clustering and t-SNE plots of pan-cancer analyses are primarily driven by cell of origin [3,10,15,16]. This finding includes the observations that (a) colon cancer and rectal cancer are indistinguishable based on the usual transcriptomic profiles, and (b) esophageal cancers split into two groups, one of which clusters with stomach adenocarcinomas and one of which clusters with head and neck squamous cell cancers. These exceptional cases are consistent with the known biological relations between the tissue types [17,32,33]. These findings also suggest that gene expression patterns that were already established in those cells before they became cancerous persisted after transformation. Moreover, those existing expression patterns appear to be the strongest signal in the data, dominating expression signatures arising from specific gene mutations in specific tumors.

Our second finding (Figure 2B,D and Figure 3B) is that using Jaccard distance metrics computed from binary mutation data does not lead to a clear clustering result. There are few clear boundaries, and very little in the way of well separated clusters. The silhouette width plot (Figure 3B), in particular, shows that about half the samples are not well clustered, and may be more similar to other clusters than the one to which they are assigned.

In spite of the apparently low quality of many of the mutation clustering assignments, a deeper look at the genes associated with the clusters suggests that using the mutation data for clustering enables the discovery of patterns that cannot easily be found by any other unsupervised method. For example, the samples in cluster MU.5 have many more mutated genes on average than any other cluster. Note that MU.5 contains the lavender samples in the lower left corner of Figure 2B, which is the cluster most clearly separated from everything else. This observation is consistent with the silhouette width plot in Figure 3B, where all samples in MU.5 appear to be well clustered into the proper group. The cancer types contributing to MU.5 include 81 STAD, 56 COAD, 13 HNSC, and fewer than five samples from any of the other three cohorts. Higher mutation rates are often associated with microsatellite instability (MSI) [34,35,36], a phenomenon that is more common in stomach cancer and colon cancer than in other digestive tract cancer types [37,38]. Thus, clustering based on the binary mutation patterns may have detected a set of MSI cancer samples.

At the other extreme, mutation cluster MU.6 has no strongly associated mutated genes and, in fact, includes 25 cases that have no mutated genes. It is, therefore, not surprising that almost all samples in MU.6 are marked as poorly clustered in Figure 3B. All four of the remaining clusters are strongly associated with at least one or two mutated genes:

- The *APC* gene is mutated in almost 75% of the cases in MU.1 (where 70% of the cases are either COAD or READ). *APC* has long been known as one of the most frequently mutated genes in colorectal cancer [39].
- The *SMAD4* gene is mutated in more than 98% of the cases in MU.2. *SMAD4* is known to be a common mutation in pancreatic cancer [40,41], colorectal cancer [42,43], and gastric cancer [38].
- The *TP53* gene, which is known to be the most commonly mutated gene across cancer types [44], is mutated in almost 78% of the cases in MU.3 and 91% of the cases in MU.4. Both of these rates are higher than the overall level of about 62% of mutated cases in this data set.
- By the chi-squared test, MU.4 is also strongly associated with two other mutated genes, *KRAS* and *CDKN2A*. *KRAS* mutations are known to be relatively common in pancreatic cancer and colorectal cancer [45,46]. *CDKN2A* is known to be mutated in both pancreatic cancer [47] and head and neck cancer [48]. The co-occurrence of these two mutations in pancreatic cancer has also been reported previously [47].

It is not clear why clusters are so poorly separated when using the binary mutation data. Our first thought was that it might be a consequence of the presence of “passenger” mutations that blur the boundaries. However, the fact that the highly mutated MSI cases define the most clearly identifiable cluster may contradict that notion. It also appears that there are overlaps between the relatively small sets of most frequently mutated genes in the other clusters. Among the four clusters that are strongly associated with only one or two genes by the chi-squared test, *TP53* is one of the five most commonly mutated genes in all four clusters (Table 2). *KRAS* is frequently mutated in three clusters; *APC* in two; and *CDKN2A* in two. It is possible that the differences between mutation clusters may be more subtle, caused by less common mutations.

Next, we turn to the clusters that were found when using Jaccard distance on the binary Newmanized transcriptomic data. Like the clusters found from the usual transcriptomic data, these clusters are well defined, have sharp boundaries, and are cleanly separated, both visually and by silhouette width (Figure 1A and Figure 3A). One of the most interesting findings from this cluster analysis is that colon cancer (which forms part of cluster NO.1, in the lower right corner of Figure 2A,C, along with some cases of esophageal cancer) can be completely separated from rectal cancer (which forms part of cluster NO.2, in the lower left corner of Figure 2A,C, along with some cases of pancreatic cancer). Previous analyses based on the usual transcriptomic profiles have repeatedly found these two cancer cohorts to be indistinguishable [3,10,15]. (Also see the NIH website https://stagetestdomain3.nih.gov/news-events/nih-research-matters/colon-rectal-cancers-surprisingly-similar (accessed on 27 April 2026)).

Paschke and colleagues [49] have argued that the term “colorectal cancer” should be avoided, and the two cancer types should be regarded as distinct entities. Our use of Newmanization gives strong support to their arguments, and it may provide tools to better understand the molecular differences between colon and rectal cancer. The mutation patterns, by themselves, are unlikely to fully explain the differences. Four of the five most frequently mutated genes are on both lists (Table 2). *APC* (56% vs. 49%) and *FAT4* (21% vs. 13%) mutations are more common in NO.1; *TP53* (71% vs. 61%) and *KRAS* (54% vs. 33%) mutations are more common in NO.2. At lower (but statistically significant) levels, NO.1 cases are more likely to have *BRAF* or *PI3KCA* mutations, and NO.2 cases are more likely to have *NRAS* or *SMAD4* mutations.

Our gene enrichment analysis of the differences between clusters NO.1 and NO.2 were driven by pathways involving the extracellular matrix, cell adhesion, and, in particular, collagens and integrins. Rectal cancer and colon cancer have previously been reported to express different collagens, with implications for tumor progression and aggressiveness [50,51,52]. Rectal cancer has also been reported to have higher expression of some integrins [53]. Those results were based on direct comparisons of colon cancer and rectal cancer, using gene expression microarrays [50,53] or immunohistochemistry [51,52].

We also performed a gene enrichment analysis comparing the two NewmanOmics clusters that contained esophageal cancers. Our findings are consistent with previous reports that APOBEC mutation signatures are more common in esophageal squamous cell cancers than esophageal adenocarcinomas [54,55]. It is also known that differences in expression of HLA Class II molecules and rates of histone modifications are related to prognosis in esophageal cancer, possibly with differential effects between adenocarcinoma and squamous cell carcinoma [56,57,58]

Our study does have several limitations, many of which are direct or indirect effects of relying solely on retrospective TCGA data. First, we do not have independent, external validation of the results reported here. However, it is often difficult to find adequate data sets and algorithms to validate clustering results. Second, the number of normal samples within cohorts is sometimes limited. As we mentioned previously, our approach of using the loess algorithm to fit a smooth curve relating the standard deviation to the mean expression of genes within a normal cohort seems to make this situation less problematic than other methods. The use of an arbitrary threshold (in units related to a standard normal distribution) might be a problem in some applications of the Newman bank statistic for uncovering differentially expressed genes, but this could potentially be improved by incorporating other tools (possibly simulations) to estimate the false discovery rate. Finally, the clinical correlates of patients samples in the TCGA data set are quite limited, and so we are at this point unable to tell if the new groupings we discovered are clinically relevant. Instead, we have to settle for showing that they are at least related to gene expression patterns and to mutation patterns, and through those, to biological patterns found with gene enrichment.

## 5. Conclusions

Clustering using either the binary mutation data or the binary Newmanized transcriptomic data yields useful insights into the relationships between different cancers of the digestive tract. Based on our study, the strength of unsupervised clustering based on mutations is that it is able to find the set of highly mutated tumors with microsatellite instability, along with a set of tumors with very few (or no) mutations. Its weakness is that it is unable to cleanly separate the remaining sets of tumors, which have overlapping mutations in well known driver genes like *APC*, *TP53*, *KRAS*, *KMT2D*, *CDKN2A*, *PIK3CA*, and *SMAD4*.

The strength of unsupervised clustering using the Newmanized data is that it identifies several well-separated clusters of tumor samples with common features in the way in which their gene expression patterns differ from normal. By removing the dominant “cell-of-origin” signal from each tumor type, we were able to use an unsupervised method to separate colon cancer and rectal cancer into distinct clusters. Gene enrichment analysis using those clusters was able to recapitulate known differences that had originally been found by supervised analyses using different technologies. We were also able to separate the esophageal cancer samples into two groups that are not the usual adenocarcinoma vs. squamous cell carcinoma split, and we were able to characterize the differences in terms of differential expression of genes that could also be found in the literature.

Future Plans. We believe that the Newman statistics will become a useful tool for understanding transcriptomic data. One next step would be to apply the idea to the task of finding genes that are differentially expressed between cancer and normal samples. This application would avoid the dichotomization step that we used here in order to be able to compare the Newmanized transcriptomic data to the mutation data. One would, as mentioned previously, have to estimate the false discovery rate, probably by studying the distribution of gene-by-gene *p*-values from *t*-tests. A more exciting potential application would be to test whether the method could be applied to paired tumor and normal samples. After all, two samples is enough to compute a crude estimate of both the mean and standard deviation of gene expression. One could still use loess to smooth those estimates and thus borrow strength across genes and compute better (nearly independent) estimates of the standard deviation. One could then test the significance of the difference in the two measurements, which, in this context, is just the range (maximum–minimum) of expression in the data set. If that idea works, it could form the basis of a notion of “personalized transcriptomics” to augment the mutation-based personalized medicine that has already begun to revolutionize the treatment of cancer patients.

## Figures and Tables

**Table 1:** Number of samples by digestive tract cancer site of origin.

	COAD	ESCA	HNSC	PAAD	READ	STAD
Cancer	279	183	500	171	92	410
Normal	41	11	44	4	10	35

**Table 2:** Top five significant genes, by cluster. Colors denote data-driven clusters as shown in Figure 2 and Figure 3. Columns contaniing arbitrary cluster numbers are denoted with #.

(A) RNA, NO Clusters			(B) RNA, MU Clusters			(C) DNA, NO Clusters			(D) DNA, MU Clusters		
RN.#	RN.Gene	RN.%	RM.#	RM.Gene	RM.%	MN.#	MN.Gene	MN.%	MM.#	MM.Gene	MM.%
1	OTOP2	100.0	1	XIST	86.2	1	TP53	61.4	1	APC	74.5
1	KRT80	100.0	1	ADH1B	82.2	1	APC	56.5	1	TP53	67.8
1	CA7	100.0	1	SFRP1	80.5	1	KRAS	33.2	1	KRAS	45.0
1	BEST4	100.0	1	COL11A1	79.2	1	PIK3CA	24.1	1	PIK3CA	26.8
1	SCARA5	99.7	1	UGT1A10	78.2	1	FAT4	21.4	1	FAT4	22.5
2	AGR2	97.5	2	MUC5B	83.3	2	TP53	71.1	2	SMAD4	98.3
2	XIST	96.9	2	DMBT1	73.3	2	KRAS	54.1	2	TP53	65.0
2	CEACAM6	95.6	2	MMP1	71.7	2	APC	49.1	2	KRAS	48.3
2	TMPRSS4	94.3	2	KRT7	71.7	2	SMAD4	17.6	2	APC	16.7
2	KRT19	93.1	2	CEACAM6	71.7	2	FAT4	13.2	2	RNF43	10.0
3	MAL	95.6	3	ADH1B	83.8	3	TP53	46.1	3	TP53	77.8
3	ESM1	90.9	3	MYOC	83.5	3	LRP1B	27.8	3	LRP1B	20.2
3	CST1	90.4	3	PLIN4	83.1	3	ARID1A	25.6	3	FAT1	12.9
3	SCNN1B	88.2	3	MAL	82.6	3	FAT4	20.9	3	KMT2D	11.0
3	PGA4	88.2	3	PI16	79.7	3	KMT2D	17.0	3	CDKN2A	9.7
4	CRISP3	93.4	4	MUC5B	89.4	4	TP53	67.2	4	TP53	90.8
4	ADH1B	91.3	4	PIGR	79.4	4	FAT1	17.0	4	KRAS	58.2
4	ADIPOQ	89.5	4	ADH1B	79.4	4	CDKN2A	17.0	4	CDKN2A	44.0
4	MUC7	89.1	4	DMBT1	75.9	4	KRAS	16.6	4	RNF213	7.8
4	GPD1	88.6	4	PLIN4	73.0	4	PIK3CA	15.7	4	SMAD4	6.4
5	ATP4A	99.5	5	CLEC3B	93.7	5	TP53	79.1	5	KMT2D	64.8
5	PGA5	98.9	5	MYOC	93.1	5	LRP1B	20.3	5	ARID1A	62.3
5	GKN1	98.9	5	SCNN1B	91.2	5	CDKN2A	15.5	5	FAT4	57.9
5	GIF	98.9	5	SCARA5	89.9	5	KMT2D	15.0	5	RNF213	50.3
5	ATP4B	98.9	5	PI16	89.9	5	NOTCH1	13.4	5	LRP1B	49.1
6	CRISP3	96.5	6	MAL	84.3	6	TP53	65.8	6	PIK3CA	22.6
6	PRH2	96.1	6	COL10A1	77.0	6	FAT1	19.7	6	ARID1A	16.5
6	MMP1	96.1	6	PLIN4	75.4	6	CDKN2A	19.7	6	CASP8	11.3
6	ZG16B	94.7	6	DMBT1	74.6	6	PIK3CA	14.4	6	TP53	10.9
6	FAM3B	94.4	6	MYOC	73.8	6	KRAS	14.1	6	FAT4	10.5

RN = mRNA features associated with clusters defined by Nemmanomics (NO); RM = mRNA features associated with clusters defined by mutations (MU); MN = mutated genes associated with clusters defined by Nemmanomics (NO); MM = mutated genes associated with clusters defined by mutations (MU).

**Table 3:** Comparison between NewmanOmics clusters and histological cell types of esophageal cancer.

	NO.1	NO.5
Adenocarcinoma	57	6
Squamous Cell Carcinoma	29	28

## Data Availability

The article is a new analysis (applying a novel method) to data from the TCGA. That data is already publicly available. Our new analysis is available as an R package at CRAN, and the actual application of that pacakge to these data is available in a public git repository that we cited.

## Supplementary Materials

The following supporting information can be downloaded at: https://www.mdpi.com/article/10.3390/cancers18091427/s1, Table S1: Results of ANOVA and Chi-Squared analyses to find differences between Newmanized clusters. Stored as an Excel spreadsheet with 20,476 rows and 28 columns. Table S2: Results of gene entrichment analysis of each Newmanized cluster based on genes stronly associated with the cluster.
